# Bias in artificial intelligence algorithms and recommendations for mitigation

**DOI:** 10.1371/journal.pdig.0000278

**Published:** 2023-06-22

**Authors:** Lama H. Nazer, Razan Zatarah, Shai Waldrip, Janny Xue Chen Ke, Mira Moukheiber, Ashish K. Khanna, Rachel S. Hicklen, Lama Moukheiber, Dana Moukheiber, Haobo Ma, Piyush Mathur

**Affiliations:** 1 Department of Pharmacy, King Hussein Cancer Center, Amman, Jordan; 2 Department of Medicine, Morehouse School of Medicine, Atlanta, Georgia, United States of America; 3 Department of Medicine, St. Paul’s Hospital, University of British Columbia, Dalhousie University, Vancouver, British Columbia, Canada; 4 Massachusetts Institute of Technology, Cambridge, Massachusetts, United States of America; 5 Department of Anaesthesiology, Atrium Health Wake Forest Baptist Medical Center, Winston-Salem, North Carolina, United States of America; 6 Perioperative Outcomes and Informatics Collaborative, Winston-Salem, North Carolina, United States of America; 7 Outcomes Research Consortium, Cleveland, Ohio, United States of America; 8 Research Medical Library, University of Texas MD Anderson Cancer Center, Houston, Texas, United States of America; 9 Department of Anaesthesia and Critical Care Medicine, Beth Israel Deaconess Medical Center, Boston, Massachusetts, United States of America; 10 Department of Anaesthesia and Critical Care Medicine, Cleveland Clinic, Cleveland, Ohio, United States of America; The University of Melbourne, AUSTRALIA

## Abstract

The adoption of artificial intelligence (AI) algorithms is rapidly increasing in healthcare. Such algorithms may be shaped by various factors such as social determinants of health that can influence health outcomes. While AI algorithms have been proposed as a tool to expand the reach of quality healthcare to underserved communities and improve health equity, recent literature has raised concerns about the propagation of biases and healthcare disparities through implementation of these algorithms. Thus, it is critical to understand the sources of bias inherent in AI-based algorithms. This review aims to highlight the potential sources of bias within each step of developing AI algorithms in healthcare, starting from framing the problem, data collection, preprocessing, development, and validation, as well as their full implementation. For each of these steps, we also discuss strategies to mitigate the bias and disparities. A checklist was developed with recommendations for reducing bias during the development and implementation stages. It is important for developers and users of AI-based algorithms to keep these important considerations in mind to advance health equity for all populations.

## Author summary

Though artificial intelligence (AI) algorithms were initially proposed as a means to improve healthcare and promote health equity, recent literature suggests that such algorithms are associated with bias and disparities. Therefore, we outline the various elements of potential bias in the development and implementation of AI algorithms and discuss strategies to mitigate them.

## Introduction

As healthcare continues to rely on technological innovation, the use of data-driven prediction algorithms and models is becoming more widely adopted in our society. Prediction algorithms and models use various types of data such as patient-specific demographics and disease characteristics to estimate the probability of having (diagnostic prediction) or developing (prognostic prediction) a particular disease or specific outcome [1].

Prediction algorithms are not new to healthcare, as we have seen numerous prediction scores and tools developed over several decades for various clinical conditions and settings, such as those to predict fall in elderly patients, mortality in intensive care units, chronic kidney disease, cardiovascular disease, and many others [1–7]. Historically, these models were scoring systems developed from small datasets and prioritized parsimony for ease of clinician use at the bedside. However, the availability of larger population datasets from electronic health records and computational power have facilitated analysis using methods such as artificial intelligence (AI). AI-based algorithms tend to incorporate a variety of conventional statistical methods and intensive computational machine learning methods to help understand patterns and associations within high-dimensional, nonlinear, and multimodal data [8]. Moreover, with the advances in precision medicine, AI algorithms may assist in further enhancing personalized medicine and optimal care [9].

Literature has demonstrated how AI can revolutionize healthcare through its ability to perform various clinical tasks with a comparable or arguably even faster and with greater precision performance to that of humans [10–12]. In addition, AI was proposed as a tool that could expand the reach of quality healthcare to underserved areas and improve health equity worldwide [12,13]. However, recent studies have raised concerns about biases within these algorithms that could lead to health disparities, and potentially harm [14–16]. A study by Obermeyer and colleagues further emphasized this important point by demonstrating racial bias in an algorithm widely utilized in the United States healthcare system [17]. The algorithm utilized healthcare expenditure to identify patients in need of additional care. Though expenditure may be an effective proxy for severity of illness and thus the need for additional healthcare, the authors demonstrated that utilizing the algorithm would reduce the number of Black patients needing additional care by more than half.

Understanding sources of bias within AI-based prediction algorithms as well as identifying strategies to mitigate the potential disparities are critical steps towards the advancement of health equity and human rights. The aim of this review is to highlight the major sources of bias within each step of developing AI algorithms in healthcare and to discuss strategies to reduce bias and disparities. Given the extensive literature on bias in AI, this review does not aim to provide a comprehensive description of what has been published on sources of bias and strategies to mitigate but to rather highlight the main elements under each section and provide a few major examples.

### Bias in the development of AI algorithms

**Fig 1:**
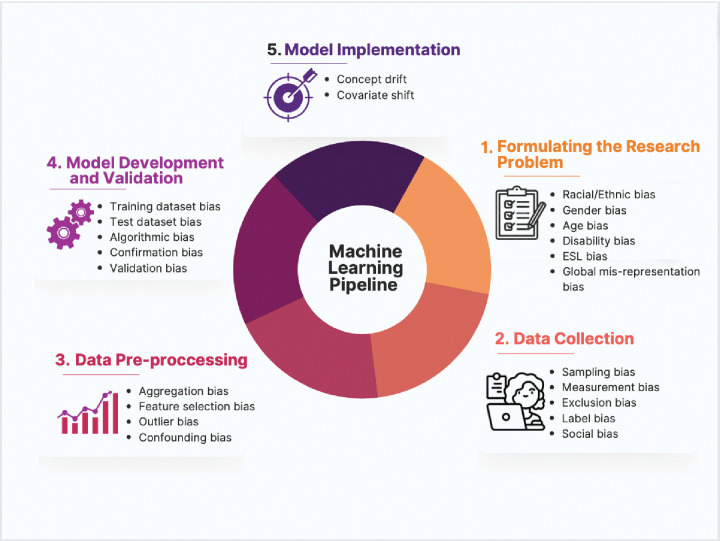
Sources of bias that may contribute to health disparities within each step of developing an AI-based algorithm.

Development of AI prediction algorithms consists of several stages, each of which may contribute to bias [18,19]. The following outlines the steps of developing AI-based algorithms and the sources of bias that may contribute to health disparities within each step (Fig 1).

### 1. Formulating the research problem

The first step in developing AI algorithms involves defining the problem(s) needing to be addressed. In formulating the research problem, it is important to evaluate the purpose and relevance of the proposed algorithm. Prediction algorithms should address questions that are clinically relevant and meaningful as well as have an impact on clinical practice [18,19]. For example, though it is important to predict illnesses, such as sepsis, the algorithm should produce actionable output that ultimately links to clinical decision-making [18,19]. Furthermore, in this step, if the problems identified were not formed with inclusivity in mind from inception, health disparities could arise from the generated tools, being restricted mostly to specific clinical problems that are relevant to a subset of patients.

Race, sex, and disability status are inequities that may determine which health-related problems are prioritized and funded, and ultimately what research, including those related to AI, is produced. Such biases would result in identifying research questions/problems that are in favor of a segment of the population, regardless of the burden of the disease. An example of racial bias is seen with cystic fibrosis (CF), which is more common in White patients and sickle cell disease (SCD), which is more common in Black patients. The most recent statistics reported approximately 40,000 patients living with CF in the US [20] and 100,000 patients with SCD [21]. Nevertheless, CF receives over 3 times more funding per affected individual from the US National Institutes of Health, compared to that for SCD, as well as hundreds of times more private funding [22]. A similar observation was reported when evaluating sex disparity and the allocation of research funding for diseases that are female dominant compared to those that are male dominant [23]. In about three-quarters of the cases where a disease was mostly dominant in one gender, the funding pattern favored males. In addition, the disparity between funding and burden-commensurate funding was about twice as large for diseases that were more dominant in males than females.

Though the recognition of racial and sex bias has increased over the years, and efforts have been generated to reduce such disparities, disability bias is less recognized and more difficult to mitigate. Disability diseases include a wide range of functional and mental diseases and there is variability in how the diseases are defined, as well as whether they are clearly documented in the patients’ medical records. Therefore, interest in conducting research and developing clinical tools for patients with disabilities remains suboptimal [24].

At a global level, bias is seen in the underrepresentation of international health problems in research priorities that are funded and studied. Such misrepresentation has been highlighted by the World Health Organization (WHO) 10/90 gap, demonstrating that the majority of research dollars and priority goes to only 10% of the global population [25]. This results in having most research, including those related to AI, address health problems that are relevant to a smaller segment of the global population.

### 2. Data collection

The data used to develop prediction algorithms is a major factor contributing to various types of bias. Sampling bias, one of the most common types of data bias, arises when the data used for developing AI algorithms is obtained from patient cohorts that are not representative of the entire population for which the system is intended to be used [26]. An example of this includes an algorithm developed to predict acute kidney injury (AKI) using clinical data from the US Department of Veteran Affairs [27]. Though the dataset used to develop the algorithm was large and diverse, containing data from over 700,000 patients from multiple centers, it was predominantly made up of data from older non-Black men; the average age was 62 years old and over 90% were males. This could affect the performance of the developed algorithm, as it may not be as reliable when used to predict AKI in younger female patients and in ethnicities that were not represented in the data.

Measurement/classification bias is another common type of data bias. This can occur when patients receive different care or are incorrectly diagnosed based on sociodemographic factors reflecting practitioner bias [28]. For example, women are less likely to receive lipid-lowering medications and procedures in the hospital compared to men, despite being more likely to present with hypertension and heart failure [29]. Therefore, if a model is developed based on such data, lipid-lowering agents would be recommended more for men, erroneously considered as having a probability of cardiac disease that is higher than women. Another example involves faulty measurements with commonly used devices such as pulse oximeters, thermometers, and sphygmomanometers [30]. Measurements of the pulse oximeter are known to be affected by the patient’s skin color, which leads to the device systematically overestimating oxygen saturation levels in non-White patients [30]. Accordingly, AI prediction algorithms that incorporate pulse oximetry as a main feature may contribute to health disparities even if the training dataset had adequate representation of Black patients.

Another bias encountered in data collection is label bias, which is seen when the outcome variable is differentially ascertained or has a different meaning across groups [31]. An example is a prediction algorithm developed to target cancer screening in patients with high rates of cancer. Communities in which cancer screenings are frequently performed will have inflated incidences compared to underserved populations that would consequently be “labeled” as having a lower incidence of cancer due to lesser screening in those areas. Since screening is not an accurate reflection of the incidence of cancer, this in-turn would result in a biased algorithm that targets screening to over-served communities, leading to further health disparities [31].

Bias due to missing data may also be encountered in the data collection step, which can produce AI algorithms that do not account for underrepresented populations. For example, countries such as Canada and France do not record race and ethnicity in their national health databases, making it difficult to account for less represented groups that may have different outcomes compared to the overall general population [22]. As mentioned earlier, there is also lack of data on disability in most datasets. The lack of such data limits the ability of researchers to understand the impact of disability on outcomes, generates algorithms that do not incorporate disability, and ultimately contributes to the exclusion of disabled people from discussions and policies that are data driven [24,32].

### 3. Data preprocessing

Preprocessing of the data refers to transforming patient-related raw data to a readable and structured digitized format that is ready for analysis. It involves analytical data manipulations such as imputations of missing values, selecting highly predictive variables, and aggregation [19]. It is crucial to ensure that these techniques account for factors that may contribute to bias and health disparities in the developed algorithms.

Aggregation may result in bias when a “one-size-fits-all” model is used for groups with different conditional distributions [33]. Hispanics, for example, tend to have higher rates of diabetes and diabetes-related complications compared to Whites [34]. Using AI-based algorithms may help diagnose and monitor diabetes in Hispanic populations; however, it can also lead to aggregation bias if the models are not sensitive to the fact that there are varying Hispanic ethnic groups (e.g., Mexicans, Puerto Ricans). If these issues are left unaddressed during this stage, the algorithm would be developed with biased data and will either have an overall poor performance or perform properly solely for the majority of the represented population.

Managing missing data and outliers is another challenging aspect during this stage. The most common approaches used to address this are complete case analysis or mean imputation [35]. With such approaches, patients with values that are missing or outliers on any of the variables are deleted from the analysis (complete case analysis) or their values are replaced by mean estimates based on the remaining data (mean imputation). Though this would facilitate the process of analyzing the data, it does not acknowledge the fact that such findings may reflect the diversity of patients. For example, weight may not be available in patients with disabilities and wheelchair users. Similarly, extreme values of weights may be seen more commonly in certain patient populations such as obesity among Black patients and lower weights in patients with limb amputation or terminal illnesses.

During this stage, the features/variables that are selected for incorporation in the model may also be a source of bias. An example for this type of bias may be encountered with prediction algorithms designed for the early screening of sepsis. The Surviving Sepsis Campaign guidelines recommend the use of machine learning algorithms that utilize scoring systems for the early screening of patients [36]. Since the Sequential Organ Failure Assessment (SOFA) score is recommended in the guidelines, it is very likely that algorithms would incorporate this score as one of the features/variables. However, several studies have demonstrated suboptimal performance of the SOFA score among various patient populations such as Black patients, female patients, and patients with disabilities [37–40]. Such findings suggest potential health disparities when utilizing AI-based algorithms that incorporate the SOFA score to guide clinical decision-making and triage of certain patient populations with suspected sepsis.

### 4. Development and validation of AI-based algorithms

Once the dataset has been transformed to a computer-readable format, it is typically split into training, test, and validation datasets [41]. The algorithm is built from the training set, while the test and validation sets are left for accuracy measurement and the validation of the developed model [41]. It is important to recognize that not all analytical methods work for all questions of interest, and some may impose health disparities among certain patient populations due to less representation or socioeconomic factors that impact the type of data available for analysis.

Overfitting is a common problem encountered during the validation of the model which may significantly impact its generalizability and contribute to bias among underrepresented groups of patients [42,43]. In the case of overfitting, the model would demonstrate very high performance when tested on its own dataset, but poorly when applied to other populations or settings. In a study that evaluated the methodological quality of 152 studies on prediction models developed using machine learning techniques, only half of the included studies examined potential overfitting of the models using appropriate strategies [43].

AI-based prediction algorithms are often criticized as being “black-box” models since the model may perform its own interpretation of various features and data to make a given prediction [41]. This raises significant concern about health disparities among minority populations who are typically less represented in these datasets as it is very likely that the algorithm may generate biased interpretations of the available data as well as any outliers or missing data for such patient populations.

### 5. Model implementation

It is not uncommon for algorithms to perform well when tested and validated, but to do poorly once implemented in the real-world or later in its lifespan. Therefore, after implementation, assessment of the algorithms needs to continue throughout the entire lifespan [18]. Continued acceptance among various clinician groups based on usability, feasibility, and generalizability are important parameters to measure successful deployment of the AI algorithms.

A well-known example of a model that demonstrated major flaws after its implementation is one which we described earlier by Obermeyer and colleagues [17]. When evaluating a widely utilized prediction model that predicted the need for healthcare among patients, significant racial and socioeconomic bias were reported. The model utilized healthcare expenditures as a proxy for the need of healthcare. While this may appear to be an appropriate measure, it was associated with health disparities. Remedying these disparities was found to increase the percentage of Black patients receiving additional care from 17.7% to 46.5% [17].

On the other hand, the model may perform well once implemented but subsequently demonstrates a decline in performance during its lifespan. A common reason for such decline is data drift, which is seen when the population characteristics on which the model was developed is different from the population characteristics on which the model is applied [44]. One type of data drift is covariate drift, which refers to a change in the distribution of the independent features between the test and training data. In such cases, models would perform worse on the testing data compared to the training data, making them poorly generalizable. Such changes are likely to occur due to temporal or geographical differences in populations. This can be problematic when predicting the onset of life-threatening diseases such as sepsis [44].

Another type of data drift is concept drift, a change in the relationship between the predictor and predicted variables. An example includes a diagnostic algorithm that uses images of patients’ skin to detect skin cancer possibly failing in the summer when more people with more sun exposure have a different color tone, compared to the skin tones used in the training data [45].

### Strategies to mitigate bias in AI-based models

While evaluating diverse sources of bias in AI algorithms is an important first step, it is essential that we identify strategies to mitigate bias during the development, validation, dissemination, and implementation of algorithms. A number of comprehensive frameworks and checklists have been developed, such as the Translational Evaluation of Healthcare AI (TEHAI) [46], the DECIDE-AI [47], the Consolidated Standards of Reporting Trials-Artificial Intelligence (CONSORT-AI) [48], the prediction model risk of bias assessment tool (PROBAST) [49], and the Checklist for AI in Medical Imaging (CLAIM) [50]. However, the goal of such tools has been primarily to guide authors and reviewers in reporting and evaluating AI algorithms but not specifically address biases that may contribute to health disparities.

Dankwa-Mullan and colleagues [51] provided a proposed framework to integrate health equity, racial justice, and the principles of ethical AI in the development lifecycle of AI algorithms. Several recommendations were provided to ensure that health disparity concerns are assessed in all steps of the AI development cycle. The recommendations included assessing the needs, describing existing workflows, defining target state, acquiring infrastructure to develop the AI system, implementing the system, as well as monitoring, evaluating, and updating the system. However, despite the importance of such a framework, the recommendations were primarily focused on the racial disparities and did not address other underrepresented patient populations in which health disparities may arise from AI algorithms.

An open-source toolkit, the AI Fairness 360, is available to help AI researchers examine, report, and mitigate discrimination and bias in machine learning models throughout the AI development lifecycle [52]. It includes a comprehensive set of metrics for datasets and models to test for biases, explanations for those metrics, and algorithms to mitigate bias in datasets and models.

In 2021, the WHO released its first guidance on the ethics and governance of AI in healthcare [53]. Though it was clearly stated that AI holds great promise for improving healthcare worldwide, the guidance emphasized that this can only be achieved if the ethics and human rights are put at the center of its design, deployment, and use. The guidance indicated that the AI systems should be designed to reflect the diversity of socioeconomic and healthcare settings. Training and capacity building, as well as community engagement and awareness should also accompany this.

In this section, we propose strategies to mitigate bias in AI-based algorithms within each of the steps we discussed earlier. The backbone to address bias in AI models is to start with an inclusive and diverse team to work on all steps. Table 1 describes a proposed bias mitigation checklist that could be used to evaluate an AI algorithm during its development and implementation. These should be addressed at the stage of study design and vetted by research ethics boards and peer reviewers alike.

**Table 1:** A checklist to aid in mitigating bias during the development and implementation of AI algorithms.

Source of bias	Bias mitigation checklist question(s)	Action plans
**Framing the problem**	• Will the algorithm result in unintended consequences to certain groups of patients due to its hypothesis? • What subgroups make up the population? • Has diversity been encountered? • Which groups may experience potential training data errors and disparate treatment?	• Determine the availability of diverse patient populations and characteristics that support the hypothesis prior to data collection. • Engage diverse domain experts, multidisciplinary teams, and community members.
**Data sources**	• What data sources were used to develop the model? • Was there any sample size bias? • Is the data accurate and reliable? • Is there any inaccessible data? • Were the generated prediction algorithms based solely on electronic health records? • Are there any sources of sample, measurement, or label bias?	• Use publicly available datasets that could increase the diversity of the patient population used to develop the prediction algorithm. • Identify specific proportions of the patient population or features for the proposed hypothesis.
**Data preprocessing**	• Does the model account for preprocessing bias? • Were all input variables defined? • Were variables measured consistently across all subgroups? • Were there any differences in the subgroups that might affect the outcome(s)? • Were there any criteria used to mitigate preprocessing bias?	• Set well-defined input variables. • Use literature-recommended preprocessing bias mitigation techniques such as imputations, feature/variable selection, and aggregation.
**Model development**	• Were de-biasing techniques adopted to prevent algorithmic bias? • Was there a clear method defined for developing the algorithm? • Were the appropriate analytical methods used?	• Maximize the model’s prediction accuracy through using de-biasing techniques. • Explain the model’s methodology in a transparent, interpretable, and reproducible way.
**Model validation**	• Was the model internally and/or externally validated? • Was there any difference in performance between the developed and validated subgroups?	Report any differences in the model’s performance and adjust decision thresholds based on the values of sensitive features
**Model implementation**	• Will the model implementation cause disparities across certain subgroups? • Will the model be monitored and assessed for model drift?	Document how the model’s performance will be monitored and managed for disparities

### 1. Correct framing of the problem

To mitigate algorithmic bias in AI, the key is to create the model hypothesis and concept and to clearly define the desired outcomes from the algorithm. The first step starts with ensuring diversity and representation in the research team. Such diversity requires not only the inclusion of the clinical domain experts and data scientists, but also key stakeholders, members from underrepresented populations, and end users [51]. The diverse team should be considered at the start with framing the problem and generating the hypothesis and should go all the way to implementations of the model among diverse populations, while evaluating performance, generalizability, and utility. When framing the problem for a prediction model, one would need to identify the research question, population(s) of interest, predictors/variables, and endpoint (i.e., outcome of interest). AI developers must consider diversity in the model design while framing the hypothesis to avoid unintentional bias towards certain groups of patients [18]. The key question that developers must always keep in mind is, “will the algorithm result in unintended consequences to a specific group of patients due to its hypothesis?” To address this question, the following should be considered: the problem setup (i.e., how much data is available at present and the complexity of the idea), experience (i.e., engaging diverse domain experts and multidisciplinary teams), patient demographics and socioeconomic status, as well as engaging with communities to understand the various experiences and potential bias.

### 2. Data diversification and representation

Ideally, AI developers should not rely solely on data derived from a single institution; instead, they should combine various datasets to ensure that key variables such as race, ethnicity, language, culture, and social determinants of health are captured and included in prediction algorithms to minimize bias. However, it may not be always feasible to capture data for all patient subgroups; in such cases, AI developers should identify potential missing data and classifications, as well as specific subgroups of patients or features that should have been included in the developed AI models in order to provide fair interpretations for the proposed hypothesis. On the other hand, some algorithms are designed for specific populations/settings and may not need to be broadly inclusive. In such cases, AI developers should still ensure that all subgroups within the specific population/setting are captured.

Over the past decade, governments, funders, and institutions have promoted open data sharing to provide access to a variety of data sources [54]. Though numerous publicly available datasets are currently available for AI researchers to use, most still lack diversity and the vulnerable patient populations continue to be underrepresented. In a recent review of the literature on clinical AI, Celi and colleagues reported that over half of the databases used to train models primarily reflected patients treated in the US and China [55].

Further strategies should aim to expand the availability of datasets that are diverse, inclusive, and publicly available. However, such an approach may not be feasible for many institutions, especially due to concerns about privacy and security. In addition, such initiatives would take time and require development of complex and costly infrastructures that may not be feasible in all settings. To address this, healthcare institutions, academia, industry, governmental agencies, as well as patients, should partner together to promote the development of inclusive and diverse datasets. An innovative example for such an approach is the NIH *All of Us* Research Program, which aims to advance precision medicine by partnering with 1 million diverse participants across the US [56]. The research program will include data derived from participants through surveys, physical measurements, electronic medical records, biospecimens, wearables, and links to external data sources to provide active and passive data collection.

As we start seeing more datasets available for AI researchers, it is important that there are standards to ensure the quality and representation of the datasets. To address this, the STANDING TOGETHER project was initiated to develop standards that ensure datasets for training and testing AI systems are diverse, inclusive, and promote AI generalizability [57]. New recommendations will be developed for AI datasets to determine who is represented and how this information is provided.

Another suggested strategy to enhance the generalizability and representation of the developed AI models is to share the code so that other hospitals across the globe can train and validate the existing algorithms with data collected from their local institutions [54]. This can help in adjusting the model so that it reflects the patient population that it will be used for.

### 3. Identifying sources of bias

Prior to developing the AI model, it is crucial to identify all potential sources of bias relevant to the specific purpose of the model and the target patient population/setting. However, this step is complicated by the fact that bias is integrated within our clinical practices and our healthcare systems and eventually reflected in the patient-related datasets.

Though gender, race, and ethnicity are frequently identified as potential sources of bias, other factors may contribute to bias such as age, socioeconomic differences, and geography [58]. Certain measurements derived from commonly used medical devices may also be sources of bias among patient subpopulations, such as pulse oximeters, thermometers, and sphygmomanometers [30].

There are also other sources of bias that we may not be aware of. Furthermore, most of the identified sources of bias reflect those seen in developed countries, mostly North America and Europe. Our understanding of potential sources of bias in global healthcare is limited. Therefore, the development of an AI algorithm requires a diverse team that incorporates members from various disciplines, genders, racial/ethnic groups, as well as representation from various geographical regions and cultural backgrounds to help in identifying potential sources of bias.

### 4. Managing bias in data preprocessing

The next step in bias mitigation is the preprocessing phase, which involves the preparation of data for analysis. To reduce such bias during this step, developers must be transparent about the selected training data and the various data-processing techniques utilized, such as those for aggregation and imputations. Furthermore, it is essential to define the patient demographics and baseline characteristics that are utilized in the model, such as the age groups, race, ethnicity, and gender [26]. In addition, all input variables must be well defined, measured, and equally distributed across all subgroups.

Several techniques have been suggested to mitigate preprocessing bias, including re-weighing (assigning different weights to the training data based on the categories of sensitive attributes and outcomes), suppression (removing sensitive attributes) or massaging the dataset (changing labels to remove bias), and multiple imputations [35,59]. In addition, there are various machine learning methods with built-in-capabilities for handling missing data that may be utilized [35].

### 5. Eliminating bias during model development and validation

Beyond creating diverse datasets in the preprocessing phase, the mathematical algorithms used in the model development “in-processing phase” may also result in bias. To reduce the risk of such biases, mathematical de-biasing approaches such as adversarial de-biasing or oversampling have been introduced [26]. With such techniques, the model is forced to account for underrepresented groups to achieve better performance. However, such de-biasing techniques have emerged recently in computer science and more research is necessary to demonstrate that the de-biasing was achievable.

The model validation “post-processing phase” is an additional safeguard in which one may intervene to decrease the impact of biases. Similar to drug or device trials, models need to undergo real-world testing prior to deployment to assess their performance, usability, feasibility, and adoption to ensure they are working as intended and have a positive impact on patient health. Various post-processing bias mitigation techniques and performance metrics have been proposed to adjust and reduce biases within the prediction models.

Recently, the Evidence-based Practice Center Program at the Agency for Healthcare Research and Quality (AHRQ) conducted a systematic review to identify strategies to mitigate racial and ethnic bias in healthcare algorithms [60]. Studies that described algorithms that were re-designed in response to data showing racial and ethnic disparities utilized 6 major strategies to mitigate disparities. The strategies included: removing an input variable, replacing a variable, adding one or more variables, changing or diversifying the racial and ethnic composition of the patient population used to train or validate a model, creating separate algorithms or thresholds for different populations, and modifying the statistical or analytic techniques used by an algorithm [60]. The most common approach, used in 15 of 33 studies, was to remove race. Nevertheless, the investigators could not recommend a preferred specific strategy as it is likely that the effectiveness of any approach depends on several factors related to the algorithm itself, the clinical condition, population, setting, and outcomes evaluated [60].

Furthermore, in order to assess the AI algorithm’s generalizability and reproducibility, the model should be externally validated in a different dataset, i.e., a different hospital, institution, healthcare setting, or research group than that used in training and development of the model. It is crucial to ensure that the data input for the prediction models are representative and that prediction models are tailored to the population of interest and properly validated, since a model that performs well in a certain population/setting may not necessarily have similar performance in others.

### 6. Equitable model implementation

For data that is likely to change over time, developers should document how performance levels will be monitored and managed. Reporting guidelines such as DECIDE-AI are a step in the right direction to providing guidance during the early phases of implementation of decision support systems driven by AI, which might also reduce biases during the early phase of implementation [47]. An additional solution could be to develop ways to receive and include feedback from stakeholders with various backgrounds to help in determining how well the prediction model is working.

## Conclusions

With growing evidence of bias in the development and implementation of AI-based prediction models, identifying and mitigating the sources of bias in each step is critical. However, our current understanding of bias in AI reflects no more than the tip of the iceberg. To ensure that AI technology and tools achieve their full potential of improving healthcare rather than being a source for further disparities, stakeholders, including clinicians, AI researchers, patient advocacy groups, health equity scholars, governmental agencies, as well as industry across institutions and around the globe should combine efforts to enhance the representation in the AI models.
